# Supplementary material for: Methadone in Swedish specialized palliative care—Is it the magic bullet in complex cancer-related pain?
Source: PLoS One. 2020 Apr 10;15(4):e0230845. doi: 10.1371/journal.pone.0230845 (PMC7147740; doi:10.1371/journal.pone.0230845)
Supplement: S1 Text — (DOCX) [file pone.0230845.s001.docx]

# Semi-structured interview guide

**The questions below were only initial questions, with the possibility to follow-up questions, depending on the initial response. When needed, the questions were modified to suit the actual interview situation.**

## English

Tell me about yourself and your medical background.

Tell me about your experience of treating pain over the years.

Do you have experience in dealing with difficult cases? If so, how much experience do you have? Have you developed own strategies (for complex pain management)?

What is your experience of using methadone in pain management, if any? Please evolve.

If you use methadone, what aspects do you consider before introducing it to a patient?
(*Despite the open question, recurrent responses on this question were about indications and even diagnoses suitable for methadone treatment*)

Do you think you have sufficient knowledge and experience to prescribe methadone? Describe!

Which pain mechanism(s) do you consider most important for choosing methadone?

Describe a patient who you would expect to benefit from the use of methadone for analgesia.

Regarding methadone use: how is the general knowledge level among your colleagues and how do they use methadone, according to your opinion?

How is methadone perceived among your colleagues – any positive or negative attitudes?

Would you recommend a new colleague to prescribe methadone? Why or why not?

How is the level of knowledge about methadone among other staff?

Describe any occurring prejudices about methadone that you have encountered [in colleagues, staff, patients or their next of kin].

Do you remember any case where methadone treatment resulted in an unexpectedly good pain relief?

Do you remember any case where methadone use became particularly problematic [of any reason]?

Finally, if or when pain relief is obtained in a patient with an unusually difficult pain syndrome – what does it mean, at large [to the patient, the family, the staff and to yourself]?

## Swedish

Berätta om dig själv och din medicinska bakgrund.

Berätta om din erfarenhet av att behandla smärta genom åren.

Hur stor erfarenhet har du av att hantera svåra fall? Om du har det, hur mycket erfarenhet har du? Har du utvecklat några egna strategier (för komplex smärtbehandling)?

Vilken erfarenhet har du av att använda metadon i smärtbehandling, om någon? Utveckla.

Om du använder metadon, vad överväger du innan du sätter in det på en patient?

Tycker du att du har tillräckliga kunskaper och erfarenhet för att förskriva metadon? Beskriv!

Vilka smärtmekanismer anser du är de viktigaste för att välja metadon?

Beskriv en patient som du skulle förvänta dig kan dra nytta av insättning av metadon mot smärta.

När det gäller metadonanvändning: hur är den allmänna kunskapsnivån bland dina kollegor och hur använder de metadon, tycker du?

Hur uppfattas metadon bland dina kollegor – finns positiva eller negativa attityder?

Skulle du rekommendera en ny kollega att förskriva metadon? Varför eller varför inte?

Hur ser kunskapen ut om metadon bland övrig personal?

Beskriv eventuella förekommande fördomar om metadon som du har stött på [hos kollegor, personal, patienter eller deras närstående].

Kommer du ihåg något fall där metadonbehandling resulterade i en oväntat god smärtlindring?

Kommer du ihåg något fall där metadonanvändning blev särskilt problematisk [oavsett orsak]?

Slutligen, om eller när smärtlindring erhålls hos en patient med ett ovanligt svårt smärtsyndrom - vad betyder det i stort [för patienten, familjen, personalen och för dig själv]?
