# Supplementary material for: Methadone in Swedish specialized palliative care—Is it the magic bullet in complex cancer-related pain?
Source: PLoS One. 2020 Apr 10;15(4):e0230845. doi: 10.1371/journal.pone.0230845 (PMC7147740; doi:10.1371/journal.pone.0230845)
Supplement: Attachment — Submitted filename: Response to reviewers.docx [file pone.0230845.s002.docx]

Response to Reviewers, PONE-D-20-00882

**Methadone in Swedish specialized palliative care – is it the magic bullet in complex cancer-related pain?**

Dear Editor,

Thank you for your encouraging decision letter. Please find our response below.

After this revision, we hope the manuscript will be suitable for publication in PLOS ONE.

With kind regards

Per Fürst
MD, Consultant in palliative medicine
Karolinska Institutet and Stockholm Sjukhem Foundation

All changes are marked in yellow in the revised manuscript.

**Response to Academic Editor and journal requirements:**

1. Based on the style requirements by PLOS ONE we have reviewed the manuscript, including the file naming.
2. We have included as Supporting Information, named *S1 Text*, a copy of the interview guide in English and Swedish.
3. As researchers we have the utmost respect for the value of publishing scientific data as a crucial factor for increased transparency in the research process. Unfortunately, in the context of this study, there are some important limitations that prevent us from publishing the qualitative interviews on-line.
   When planning this study, an approval from Regional Ethical Review Board of Stockholm (EPN) was an absolute requirement. According to the requirements of EPN (Dnr. 2017/2302), a finalized protocol of informed consent was to be included in the application. The exact wording in this protocol/letter of consent was *“…all results will be presented anonymously and in the form of processed and condensed data in a scientific paper. It will not be possible to identify individual persons from the material. Your personal data will be handled according to “Personuppgiftslagen” (Privacy Act) PUL 1998.204…”* These regulations have been strengthened further with the introduction of GDPR in Sweden (2019). For this reason, we are not able to publish such data on-line.

Further, the data we have collected and used as the basis for our analysis in this study are interviews with individual informants, all of whom are clinically active physicians in specialized palliative care in various locations in Sweden. The number of physicians in this medical area is limited and if you are an active physician or other healthcare professional in the area you can probably in many cases read through and, with fairly high accuracy, guess who the informants are. All in all, it is therefore our assessment that even after fairly extensive de-identification, the risk that the informants' identities could be revealed is too great to allow the interviews to be published on-line.

To try to meet the scientific requirements we therefore provide contact information to the Data Protection Representative responsible for GDPR at Stockholm Sjukhem, e-mail [dso@stockholmssjukhem.se](mailto:dso@stockholmssjukhem.se) to whom anyone can turn in case of a further request.

**Response to Reviewer 1’s comments**

Thank you for your valuable comments to which we respond below.

1. Grammatical errors are corrected as follows:
   - Line 26 now 29. The word “*imposing”* has been changed to “*affecting* methadone’s *use as an analgesic”.*
   - Line 114 now 117. The comma after *“[21)”* was removed and the first letter after the colon sign was capitalized.
   - Line 166 now 169. *“a low precision”* was changed to *“low precision”*
   - Line 180 now line 187. The word *“an”* was added.
   - Line 192 now line 199. Changed to “*types*”
   - Line 193 now line 200. “*a limited*” changed to “*limited*”
   - Line 394 now line 403. “*a convulsive state*” changed to “*muscle spasm*”
2. Thank you for requesting further presentation of data to prove the conclusion that the participants may overlook other cancer pain strategies in their enthusiasm for methadone (line 366, now line 371). We find that this improves the manuscript and data is now added in the form of a new citation in the Findings section (line 172):

*[Pain specialist:] When methadone is introduced, it sometimes implies an overuse of an unconsidered treatment, while there probably sometimes are [alternative] oncological treatments, or it is more proper to consider a spinal catheter or something else. (Informant 14)*

**Response to Reviewer 2’s comments**

Thank you for your valuable comments. Please see below for our response.

1. Capitalizations of “end-of-life” are removed throughout the manuscript.
2. Line 26 now 29. The word “*imposing”* has been changed to “*affecting* methadone’s *use as an analgesic”.*
3. Line 26 now line 30. “bone metastatic” is changed to “bone-metastatic”.
4. Thank you for this suggestion. As non-native English speakers we have carefully considered whether we should use the words *low-dose add-on, coanalgesic* or *adjuvant* to describe the use of low-dose methadone in order to achieve an analgesic effect that may appear, at least occasionally, when a low dose of methadone is added to an already ongoing opioid therapy. We have concluded that all three terms have a similar, but not identical, meaning. We have also found that the definition of "adjuvant analgesics" primarily refers to the use of drugs that have a primary indication that is other than pain (see e.g. Lussier D. Oncologist 2004. Adjuvant analgesics in cancer pain management). When it comes to methadone, there are primarily two different pain-relieving effects that we are likely to benefit from, the my-receptor stimulant effect and the NMDA-receptor inhibiting effect. Instead, we have therefore considered using the word *coanalgesic*, which occurs elsewhere in the literature (e.g. Courtemanche F. Methadone as a Coanalgesic for Palliative Care Cancer Patients. J of Pall Med 2016), but eventually concluded that we wish to keep the term *low-dose add-on* as we think it distinguishes low-dose therapy from regular or high-dose methadone therapy when used a primary analgesic.
5. Regarding sampling methods. Specialized palliative care in Sweden is an area with a limited number of active physicians. At present, there are about 100 physicians who are specialists in palliative medicine and, additionally also about 350 other physicians, also members of SFPM (Swedish Association of Palliative Medicine). We have at least superficial knowledge of each other in this community and it was therefore from this knowledge that we were able to carry out a *max variation sampling* at a national level*. Snowball sampling* was not used in this study, since snowball sampling, according to Patton (In, Patton M Q. “Qualitative Research & Evaluation Methods”, p. 237) is especially useful when a study focuses on information-rich key informants or critical cases. Our aim was to catch a broad description of the phenomenon, as we partly focused on attitudes. Therefore, we looked for both experienced and less experienced informants.
6. Lines 149-151 now lines 152-155. Thank you for directing our attention to this incongruency in the manuscript. The full paragraph was changed as follows:
   *“The initial focus of the study was to explore negative as well as positive attitudes and opinions regarding methadone. However, it soon became apparent that ~~they~~ negative attitudes were few among physicians. In fact, most of the informants were more enthusiastic about, and provided spontaneous aspects on, the benefits and practical uses of methadone.”*
7. Line 157 now 160. The words “*delineations of*” were removed.
8. We have added a sentence in the Attitudes section of the Discussion which highlights the important conclusions of the 2017 Cochrane’s review on methadone and neuropathic pain.
9. Line 351-532 now line 358. A semicolon was added.
