# Supplementary material for: Methadone in Swedish specialized palliative care—Is it the magic bullet in complex cancer-related pain?
Source: PLoS One. 2020 Apr 10;15(4):e0230845. doi: 10.1371/journal.pone.0230845 (PMC7147740; doi:10.1371/journal.pone.0230845)
Supplement: Attachment — Submitted filename: Response to reviewers.docx [file pone.0230845.s003.docx]

Response to Reviewers, PONE-D-20-00882

**Methadone in Swedish specialized palliative care – is it the magic bullet in complex cancer-related pain?**

Dear Editor,

Thank you for your decision letter. Please find our response below.

With kind regards

Per Fürst
MD, Consultant in palliative medicine
Karolinska Institutet and Stockholm Sjukhem Foundation

All changes are marked in yellow in the revised manuscript with track changes.

**Response to Reviewer 1’s comments**

Thank you for your valuable comment!

After our limitations section we now have added, in lines 463-479, a new section with the subheading “*Recommendations for future research”*.

*“There is emerging evidence that low-dose methadone as an add-on twice daily to already existing high-dose opioid treatment increases the pain-relieving effect in complex pain [14-18]. This is also the general opinion in the current study. However, so far, this is not an evidence-based approach. Randomized controlled trials would clarify the true effect of methadone as an add-on therapy and elucidate the magnitude of the effect. Further studies on the duration of analgesia and which pain types and associated diagnoses that may benefit from low-dose add-on methadone would also be of interest.*

*Moreover, there is a need of separate studies with methadone as a primary opioid. Whereas the NMDA-inhibiting effect is aimed at in a low-dose setting, methadone´s mu-receptor effect is equally desirable when using methadone as a substitute for another strong opioid. In such a setting, and with that aim, both total doses and dosing might be different from the low-dose strategies. E.g., when intravenous methadone as a primary opioid was compared with morphine in a patient controlled analgesia study for 6 days, the patients pushed the button for next dose after 3.9 hours (+/- 0.85 hours) in the morphine arm and after 3.9 hours (+/- 1.6 hours) in the methadone arm [40]. Thus, whereas a two-dose regimen is recommended in the low-dose setting today, different regimens (e.g. three-dose regimens) might be optimal when methadone is used as a primary opioid. Randomized controlled trials are needed to clarify such hypotheses.”*
